# Osteocutaneous Turn-Up Fillet Flaps: A Spare-Parts Orthoplastic Surgery Option for a Functional Posttraumatic Below-Knee Amputation

**DOI:** 10.1055/a-2033-5803

**Published:** 2023-08-24

**Authors:** Harry Burton, Alexios Dimitrios Iliadis, Neil Jones, Aaron Saini, Nicola Bystrzonowski, Alexandros Vris, Georgios Pafitanis

**Affiliations:** 1 London Reconstructive Microsurgery Unit, Emergency Care and Trauma Division, Department of Plastic Surgery, The Royal London Hospital, Barts Health NHS Trust, London, United Kingdom; 2 Limb Reconstruction and Bone Infection Unit, Emergency Care and Trauma Division, Department of Trauma and Orthopaedics, The Royal London Hospital, Barts Health NHS Trust, London, United Kingdom; 3 Trauma and Limb Reconstruction Unit, Department of Trauma and Orthopaedics, King's College Hospital Foundation Trust, London, United Kingdom; 4 Department of Medical Sciences, Medical School, University of Cyprus, Nicosia, Cyprus

**Keywords:** osteocutaneous flap, fillet flap, spare-parts surgery, below knee amputation

## Abstract

This article portrays the authors' experience with a complex lower limb bone and soft tissue defect, following chronic osteomyelitis and pathological fracture, which was managed by the multidisciplinary orthoplastic team. The decision for functional amputation versus limb salvage was deemed necessary, enhanced by the principles of “spare parts” in reconstructive microsurgery. This case describes the successful use of the osteocutaneous distal tibia turn-up fillet flap that allowed “lowering the level of the amputation” from a through knee to a below-knee amputation (BKA) to preserve the knee joint function. We comprehensibly review reports of turn-up flaps which effectively lower the level of amputation, also applying “spare-parts” surgery principles and explore how these concepts refine complex orthoplastic approaches when limb salvage is not possible to enhance function. The osteocutaneous distal tibia turn-up fillet flap is a robust technique for modified BKA reconstructions that provides sufficient bone length to achieve a tough, sensate stump and functional knee joint.

## Introduction

During clinical decision making in lower extremity limb salvage, significant segmental loss of bone either from trauma, malignancy, or chronic osteomyelitis, which may result in amputation, is a devastating event. 1 For this reason, evaluation of the most optimal functional option of reconstruction is key, with preservation of stump length playing a crucial role in below knee pathologies, which also reflects a superior quality of life. 2 Therefore, it is important to implement the principles of an orthoplastic approach by evaluating the injury itself as well as the patient as a whole. 3 In cases where limb salvage is inappropriate, below-knee amputation (BKA) may offer a swift intervention, but if bone length is inadequate, one may consider various reconstructive options, including bone transport on the principle of distraction osteogenesis, so-called the Ilizarov technique, and can be achieved with internal or external devices. There have been multiple reports of utilizing this technique for optimizing the residual stump length. 4 5 Despite successful lengthening, frequent complications were encountered including prolonged immobility from months of external fixation, soft tissue ulceration, infection, and potential reamputation.

Cases with inadequate bone length for traditional levels of amputations around the knee joint (below - BKA, through – TKA, and above - AKA), resulting in “short” or “extremely short” stumps, have been shown to negatively affect functional prosthetic outcomes including energy consumption, velocity, and cadence—reducing physiological gait efficiency. 6 Polfer et al also reported that shorter stumps in transtibial amputees results in increased metabolic cost (maximum energy consumption – VO _2 max_ ) compared with longer stump length and therefore preserving as much limb as possible contributes to energy efficiency. 7 In terms of required bone length, Burgess defined this as 15 cm distal to knee joint with Mayfield defining “short” stumps as 5 to 10 cm of bone. 8

The concept of fillet flaps is one of utilizing “spare parts” by using tissue of amputated limbs. They can provide immediate one-stage stump salvage and are a well-recognized composite flap. These flaps can be used as a donor for several tissues to effectively improve stump quality including muscle, skin, or even bone. In BKA with inadequate bone length this principle has been used to lower the level of amputation and increase length **—** 28 reported cases are reviewed and summarized in Table 1 . 6 7 8 9 10 11 12

**Table 1:** Comprehensive review of cases lowering the level of amputation with bone lengthening turn-up flaps—including case discussed herein (top) 6 7 8 9 10 11 12 13

Author (year)	Cases ( *N* = 28)	Indication	Bone fixation method	Microvascular dissection	Turn-up bone used? (cm)	Final BKA level of amputation
This case (2022)	1	Pathological tibial and fibula fractures due to chronic osteomyelitis leaving 6 cm proximal tibia	Cannulated compression screws	Yes – PTA, perforators and tibial nerve	Distal tibia (10 cm)	16 cm
Wade et al (2017)	1	Traumatic blast injury (GA IIIB) from improvised explosive device leaving 6 cm proximal tibia	Compression plate	NP	Tibia and calcaneus (two osteosynthesis sites)	NR
Polfer and Potter (2015)	3	Traumatic blast injury (GA IIIB) - average proximal tibial length (7 cm)	Compression plate	Tibial nerve dissection and neurectomy	Distal tibia (7 cm)	14 cm
Vallier et al (2012)	14	13 Traumatic injury (GA IIIB), 1 severe burn (*minimum proximal tibia length 9 cm)	Compression plate	NP	Calcaneus (7.1 cm)	14.1 cm
Morgan et al (2007)	1	Traumatic injury (GA IIIB) High energy RTA with 17 cm tibia loss, circumferential ischemic soft tissue loss	Compression plate	NP	Distal tibia	NR
Pant and Younge (2003)	5	3 traumatic injuries and 2 oncological resections	2 cerclage wire, 2 staples, 1 compression plate	NP	Distal tibia (1 incl. talus)	NR
Song et al (1994)	2	1 traumatic injury and 1 oncological resection	Compression plate	NP	Distal tibia	NR
Younge and Dafniotis (1993)	1	Chronic osteomyelitis	Cerclage wire	NP	Distal tibia (8 cm)	14 cm

Abbreviations: BKA, Below knee amputation; GA, Gustillo-Anderson; NP, not performed; NR, not reported; PTA, posterior tibial artery; RTA, road traffic accident.

We apply this concept in a patient with a significant lower limb bone and soft tissue defect which would have left a very short stump of tibia or a through knee amputation and thus likely an inferior functional outcome. The objective was to use a sensate osteocutaneous distal tibia turn-up fillet flap from the “would-be” amputated lower limb to increase the bone length. In doing so, we were able to take advantage of the “spare-parts” principle in surgery to effectively increase stump length resulting in a functional BKA. We effectively used a modified orthoplastic approach to enhance function by preservation.

## Idea

A 68-year-old noninsulin dependent diabetic man with a body mass index of 33 presented to our unit after sustaining a left open proximal tibia and fibula pathological fracture following a low energy fall from standing. Six months prior to this injury he underwent incision and drainage for a pretibial abscess of the same limb at his local hospital. At the time he was given a diagnosis of chronic osteomyelitis of his tibia diaphysis. Due to the extensive soft tissue loss following debridement, his local plastic surgery unit reconstructed the defect with a medial gastrocnemius muscle flap and split skin graft.

**Fig. 1:**
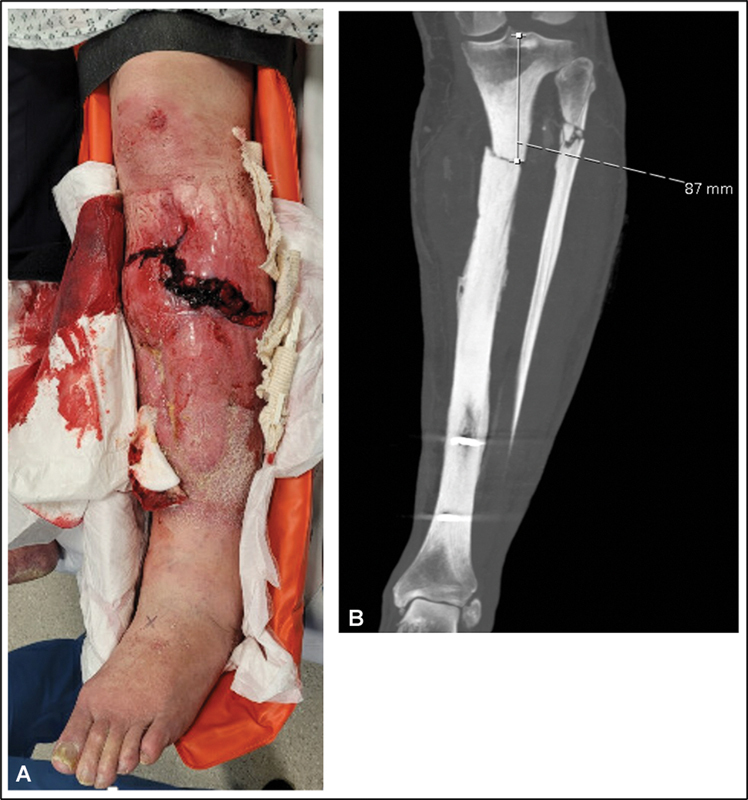
Clinical ( **A** ) and radiological ( **B** ) imaging at admission demonstrating an open proximal tibia fracture with compromised soft tissue envelope ( **A** ) on a background of chronic osteomyelitis, osteonecrosis, and a pathological tibial fracture ( **B** ).

Imaging demonstrated a pathological fracture of the proximal tibia with necrotic segmental bone loss and a poor-quality soft tissue envelope with surrounding chronic cellulitic changes not offering robust cover ( Fig. 1 ). The case was discussed in a multidisciplinary meeting between plastic and orthopaedic surgeons where it was decided that, given the comorbidities of the patient, limb salvage was not appropriate and therefore the patient should be offered a BKA. However, that would result in a very short section of healthy proximal tibia, which was suboptimal regarding the functional outcome. Furthermore, the fact that the distal third of the tibia shaft was healthy was taken into consideration. Therefore, to achieve a healthy tibial stump of adequate length, it was decided the patient would undergo a sensate osteocutaneous distal tibia turn-over fillet flap based on the posterior tibial (PT) bundle following a computerized tomographic angiogram. This was performed 7 days after injury, with informed consent from the patient.

### The Distal Tibial Osteocutaneous Turn-Up Fillet Flap

**Fig. 2:**
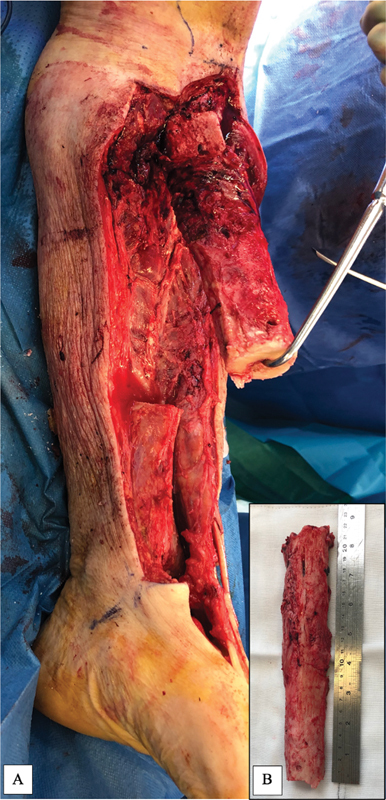
Pathological tibia bone segment in situ ( **A** ) and following resection ( **B** ) of necrotic tibia measuring 22 cm.

Intraoperatively, a pathological bone segment, measuring 22 cm, approximately 60% of the proximal tibial diaphysis, required excision due to necrosis ( Fig. 2 ). The pathological portion of the tibia was excised to healthy and bleeding bone. The fibula was excised leaving only the head and proximal 5 cm, and the ankle joint was disarticulated. The articular cartilage of the tibia plafond and the bones of the foot were also excised. Microvascular dissection was performed in layers. Sensate plantar and posterior ankle skin flap up to the midfoot was utilized and the remaining bones and soft tissue from the dorsum and toes was discarded. Peroneal and anterior tibial neurovascular bundles were tied and nerves transected. PT bundle was microscopically dissected toward its distal perforators to posteromedial skin flap and distal tibial periosteum and were preserved. The saphenous, sural, and tibial nerve along with the medial calcaneal branch were kept in continuation preserving direct innervation and therefore sensation of the fillet skin flap. Following tibial debridement and excision, a proximal 6-cm stump of healthy bone tissue and a distal portion of 10 cm of tibial shaft remained. The distal portion was reversed 180 degrees in the sagittal plane and the tibia plafond was fixed to the end of the proximal stump with two 7.3-mm cannulated compression screws, after insertion of absorbable antibiotic beads into the canal. This lengthened the tibial stump from 6 to 16 cm. The posterior skin flap was used to achieve soft tissue coverage with a 2-cm cuff of deep posterior compartment muscle supplied by the tibial bundle to prevent kinking of the turn-over flap during inset. The tibial nerve, saphenous, and sural nerves allow a sensate distal stump.

**Fig. 3:**
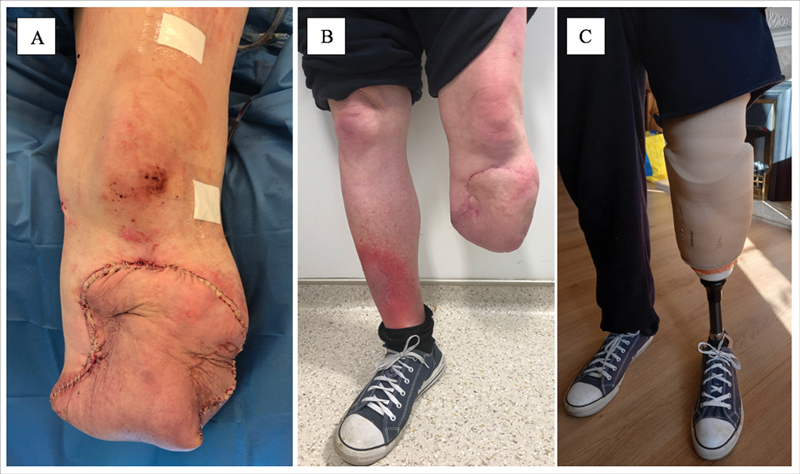
Below knee amputation (BKA) stump following fillet flap primary closure ( **A** ), at 3 months ( **B** ) and at 6 months with prosthesis fitted ( **C** ).

**Fig. 4:**
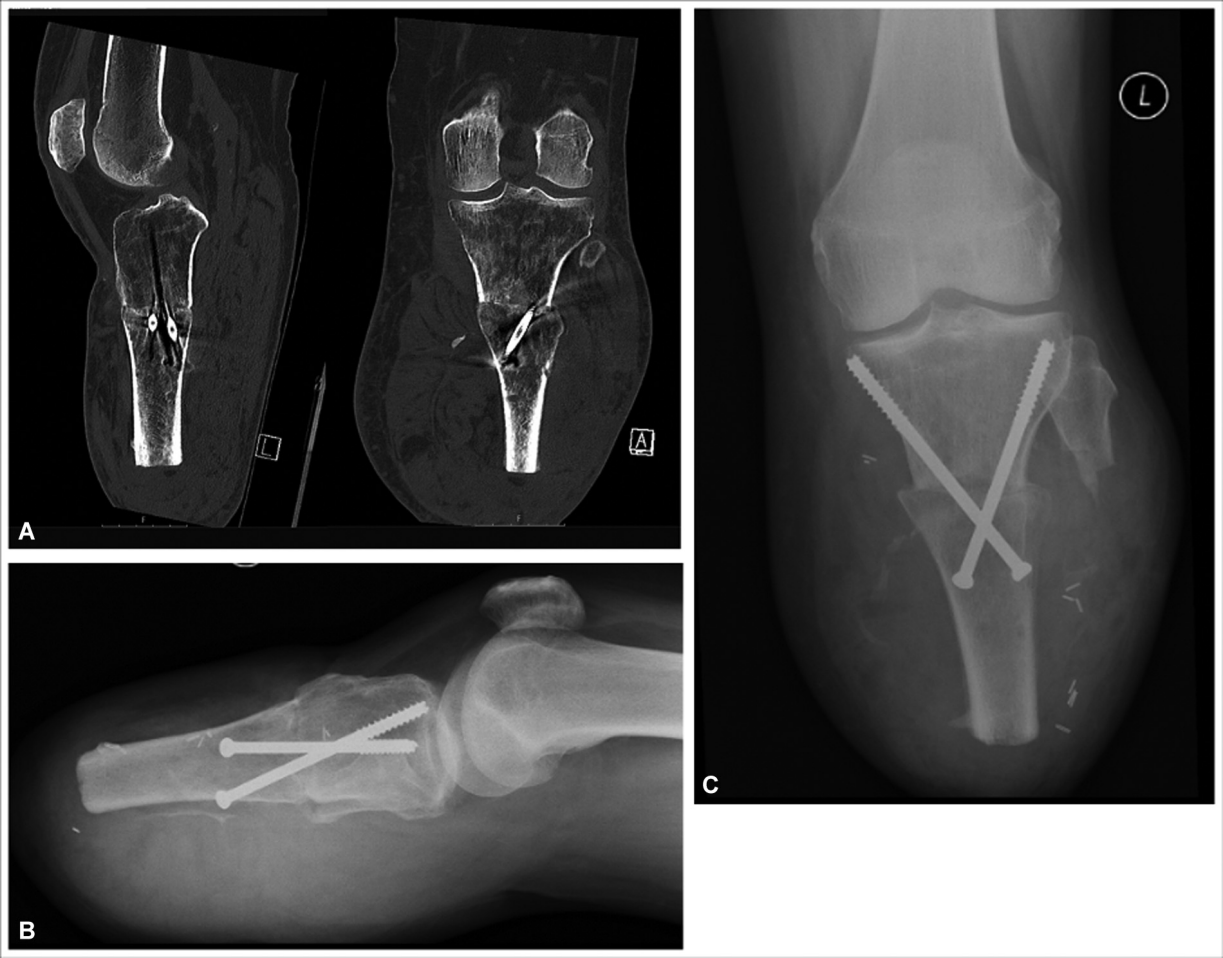
Computerized tomography ( **A** ) and plain radiographs ( **B** , **C** ) at 12 weeks demonstrating bony union.

Postoperatively, the patient was fitted with a rigid compression dressing device (ÖSSUR HF, Reykjavik, Iceland) for the first 5 days and recovered uneventfully. He was discharged home following 3 weeks of antibiotic treatment for osteomyelitis. Tissue and bone samples grew Klebsiella sensitive to co-amoxiclav and ciprofloxacin, of which he completed a 6-week course. He was seen in clinic 6 months later and demonstrated a well-healed stump with good length and preserved range of motion of the knee joint. Radiologically, the bone healed well within 12 weeks as confirmed by computerized tomography and allowed full weight bearing with a prosthesis within 6 months ( Figs. 3 and 4 ).

## Discussion

In complex lower limb infection in multimorbid patients when limb salvage is unsuitable, a short BKA stump presents several challenges including energy expenditure, prosthesis fitting, and function. The turn-up plasty demonstrates an effective way of utilizing the “spare-parts” principle in surgery when stump length is suboptimal. It effectively lengthens the proximal tibial stump using a single-stage procedure to maximize functional outcomes. Turn-up plasty has been documented in cases of trauma, tumors, and chronic infection and there is a broad variation in approaches to the flap ( Table 1 ). 6 7 8 9 10 11 12 13

In cases with large segmental bone loss and subsequent short proximal stump length the surgeon can opt for either single or multiple stage procedures to achieve bone lengthening. One may consider the Ilizarov method using distraction osteogenesis via application of an external circular frame or a lengthening nail. This has been used with success to lengthen short tibial stumps in BKAs (secondary to trauma) by over 350 mm 14 15 16 but soft tissue condition and comorbidities are important factors to consider which may deem the patient unsuitable for this procedure. Given the relatively comorbid status of this patient and the poor quality of the soft tissues due to chronic cellulitis, a lengthy distraction osteogenesis treatment was not a suitable option. These patient-specific factors also made fibula grafting unsuitable—given the tibial bone loss of 22 cm and the relative immobility that would have resulted from contralateral fibula graft harvest from the unaffected leg.

In patients more appropriate to a single-stage intervention due to comorbidities or poor compliance, one can consider bone lengthening with a fillet flap. Wu et al describes a series of successful calcaneus osteocutaneous free fillet flaps following traumatic amputations. Here, the plantar skin and deep fascia are dissected with the trimmed calcaneus. The composite flap is based on the PT artery (PTA) which is anastomosed to the popliteal artery and the calcaneus is fixed to the proximal tibial stump with Kirschner wires. This method increases bone length and achieves primary closure of the stump with durable glabrous skin from the heel pad, but requires microvascular anastomosis. 17 Twenty-eight previous reports of bone lengthening below-knee turn-up plasties without microvascular anastomosis have been summarized ( Table 1 ). These cases demonstrate the effective application of “spare parts” in surgery, despite variation in mechanism of injury, method of fixation, and patient demographic. The turn-up flaps are raised differently but they all enhance function by preservation of autologous tissue.

The main benefits to a single-stage procedure such as turn-up flaps are increased quality of life, reduced morbidity, and reduced financial burden. In addition, the advantage with turn-up plasty is single stage bone lengthening, without the need for free flap anastomoses and therefore reduced operating time. There is also preservation of plantar sensory innervation which has been shown to improve functional balance outcomes and reduce the occurrence of ulcers and pressure sores when wearing a prosthesis. 18 In this case, we performed perivascular dissection of the PT neurovascular bundle from the deep posterior compartment to maintain blood supply and sensation, while reducing muscle bulk which allows for a tension-free closure. Dissecting the PTA also enables direct visualization of its perforators, which in traumatic cases may have been injured. It is the authors' belief that the decision to dissect these neurovascular bundles or not relies on the innate skillset of the operating surgeon and should be evaluated in each individual case. The majority of reported turn-up flaps reviewed ( Table 1 ) chose not to carry out microvascular dissection of the PT neurovascular bundle.

When treating patients suffering from severe lower limb injury or infection, the “spare-parts” principle should be kept in the foreground of clinical decision making. The documented 28 cases of turn-up plasties demonstrate how effective this principle can be when applied in a single-stage operation. We believe this principle should be integrated into the algorithm of treating mangled or severely injured lower limbs, forming a bridge between limb salvage and amputation. The technique may present the answer to the clinical question of a short tibial stump, resulting in enhanced functional outcome and increased quality of adequate life years.

A limitation of this technique may present postoperatively as venous congestion of the skin paddle. To reduce likelihood of this, care should be taken to identify and preserve the distal tibial perforator vessels and where possible the great saphenous or short saphenous veins should be positioned as centrally as possible to the fillet flap width.

This case and comprehensive review of modified “spare-parts” BKAs summarizes how this technique can be used to lower the level of amputation in complex lower limb reconstruction. We demonstrate that the tibia turn-up plasty can be applied in severe bone defects caused by chronic infection as well as primary traumatic injuries. The sensate osteocutaneous distal tibia turn-up fillet flap plasty is an innovative and practical technique that use “spare-parts” principles of reconstructive microsurgery, and should make up part of the armamentarium of orthoplastic management of complex lower limb salvage versus amputation, particularly when faced with the dilemma of insufficient bone length or lowering the level of amputation.
